# Supplementary material for: Natural product P57 induces hypothermia through targeting pyridoxal kinase
Source: Nat Commun. 2023 Sep 26;14:5984. doi: 10.1038/s41467-023-41435-y (PMC10522591; doi:10.1038/s41467-023-41435-y)
Supplement: Supplementary file 1 — Supplementary Information [file 41467_2023_41435_MOESM1_ESM.pdf]

Natural Product P57 Induces Hypothermia through Targeting Pyridoxal Kinase

Ruina Wang[1†], Lei Xiao[2†], Jianbo Pan[3†], Guangsen Bao[1†], Yunmei Zhu[3], Di Zhu[1], Jun Wang[4], Chengfeng Pei[5], Qinfeng Ma[3], Xian Fu[3], Ziruoyu Wang[1], Mengdi Zhu[5], Guoxiang Wang[2], Ling Gong[2], Qiuping Tong[2], Min Jiang[2], Junchi Hu[3], Miao He[2], Yun Wang[2], Tiejun Li[6], Chunmin Liang[7], Wei Li[8], Chunmei Xia[9], Zengxia Li[1], Dengke K. Ma[10], Minjia Tan[4], Jun Yan Liu[3], Wei Jiang[1*], Cheng Luo[4*], Biao Yu[5*] & Yongjun Dang[3*]

[1]Key Laboratory of Metabolism and Molecular Medicine, Ministry of Education, Department of Biochemistry and Molecular Biology, School of Basic Medical Sciences, Shanghai Medical College, Fudan University; Shanghai, 200032, China.

[2]State Key Laboratory of Medical Neurobiology and MOE Frontiers Center for Brain Science, School of Basic Medical Sciences, Institutes of Brain Science, Shanghai Medical College, Fudan University; Shanghai, 200032, China.

[3]Basic Medicine Research and Innovation Center for Novel Target and Therapeutic Intervention, Ministry of Education, Institute of Life Sciences, the Second Affiliated Hospital of Chongqing Medical University, Chongqing Medical University, Chongqing 400010, China.

[4]State Key Laboratory of Drug Research, Shanghai Institute of Materia Medica, Chinese Academy of Sciences; Shanghai, 201203, China.

[5]State Key Laboratory of Bio-organic and Natural Products Chemistry, Shanghai Institute of Organic Chemistry, Chinese Academy of Sciences; Shanghai, 200032, China.

[6]Department of Pharmacology, College of Pharmacy, Naval Medical University; Shanghai, 200433, China.

[7]Lab of Tumor Immunology, Department of Human Anatomy, Histology and Embryology, Basic

Medical School of Fudan University, Shanghai, 200032, China.
[8]Department of Medicinal Chemistry, China Pharmaceutical University; Nanjing, 211198, China.
[9]Department of Physiology and Pathophysiology, School of Basic Medical Sciences, Shanghai Medical College, Fudan University; Shanghai, 200032, China.
[10]Department of Physiology, Cardiovascular Research Institute, University of California San Francisco; San Francisco, CA 94158, USA.
[†]These authors contributed equally: Ruina Wang, Lei Xiao, Jianbo Pan and Guangsen Bao.
*Corresponding author. Email: jiangw@fudan.edu.cn, cluo@simm.ac.cn, byu@mail.sioc.ac.cn, yjdang@cqmu.edu.cn

Supplementary Materials:

Figs.S1 to S11

Tables S1 to S3

Supplementary Figures

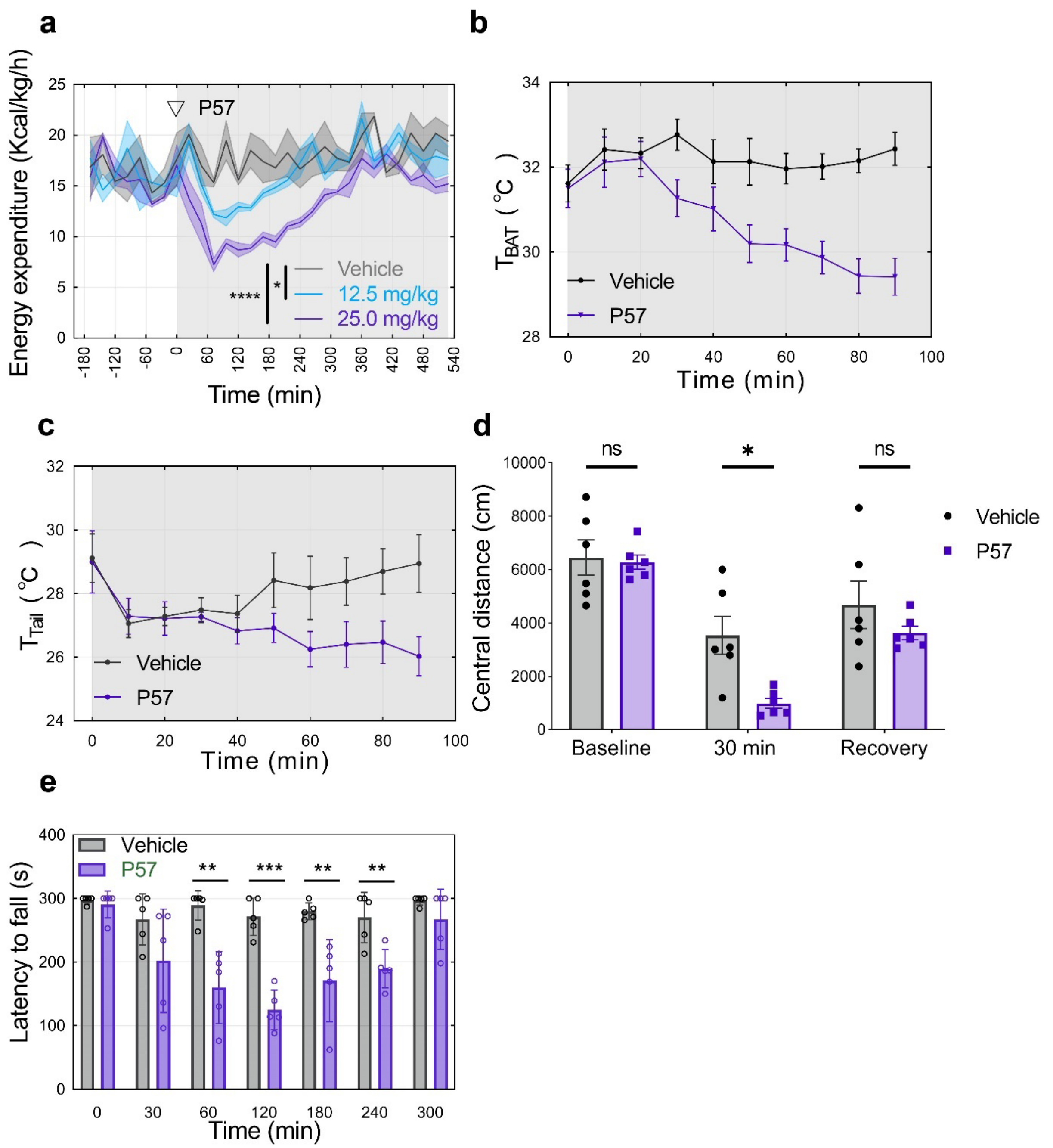

Fig. S1 Administration of P57 induces hypometabolism and decreases motor activity. a, Energy expenditure of P57-treated mice. P57 (12.5 mg/kg or 25.0 mg/kg) or the vehicle control was injected intraperitoneally into mice at 0 min (arrow); energy expenditure was calculated as the product of the calorific value of oxygen ($3.815*VO_2 + 1.232*VCO_2$), mean (n = 3 mice) ± s.e.m; significant differences between treatments were calculated using two-way analysis of variance (ANOVA), * $P < 0.05$, **** $P < 0.0001$. **b, c,** Surface temperature of BAT (b) and tail (c) of P57-treated mice. P57 (25 mg/kg) or the vehicle

was injected intraperitoneally into mice at 0 min. The surface temperature was recorded by IR digital thermographic camera (FLIR T430sc) every 10 minutes, mean (n = 6 mice) ± s.e.m. **d,** Locomotor behavior of P57-treated mice by Open field test. The motor activities at different time points (baseline before treatment with vehicle or P57, 30 minutes and 24 hours (Recovery) after treatment with Vehicle or P57) were detected. The distance moved from the center-point (central distance) was recorded continuously for 20 minutes, mean (n = 6 mice) ± s.e.m; significant differences between treatments were calculated using two-way analysis of variance (ANOVA), * $P < 0.05$. **e,** Rotarod performance of P57-treated mice. P57 (25.0 mg/kg) or the vehicle control was injected intraperitoneally into mice at 0 min. Latency to fall from a constant accelerating rotarod was recorded at different time points. Mean (n = 5 mice) ± s.e.m; ** $P < 0.01$, *** $P < 0.001$ compared to control group, as determined by student's t test (two-sided). Experiments in fig.S1 was performed under ambient temperatures at 22~24℃. Source data are provided as a Source Data file.

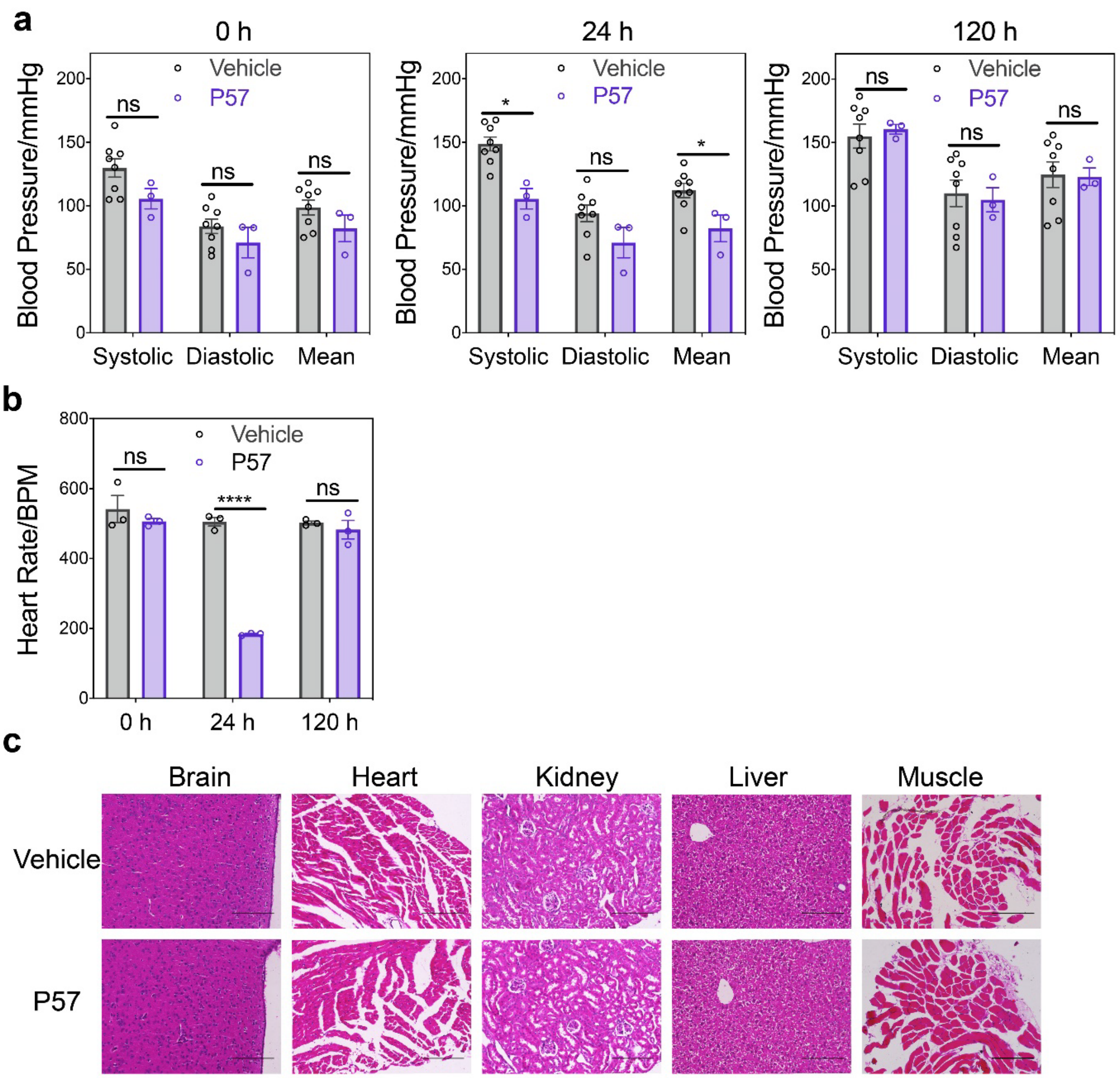

Fig. S2 Consecutive Administration of P57 induces a reversible physiological change. P57 (25.0 mg/kg) or the vehicle control was injected intraperitoneally into mice ever 3 h for 4 times. **a,** Blood pressure of P57-treated mice. Blood pressures at baseline (before treatment with vehicle or P57), 24 h and 120 h after first administration of vehicle or P57 were detected by the Coda Non-invasive blood pressure system. Mean (n = 8 for vehicle control group, and n=3 for P57 group) ± s.e.m.; * $P < 0.05$ compared to control group, as determined by student's t test. **b**, Heart rates of P57-treated mice. Heart rates of P57 at baseline (before treatment with vehicle or P57), 24 h and 120 h after first administration of vehicle or P57 were detected and analyzed by the Labchart software. Mean (n = 3 mice) ± s.e.m.; **** $P < 0.0001$ compared to control group, as determined by student's t test. **c,** Histology of tissues of P57-treated mice. After 72 h relative to the first injection of P57, the core temperature of mice have been normal (36.38 ± 0.27 °C) and tissues were

taken. Whole regions in the brain, heart, kidney, liver and soleus muscles prepared from mice received P57 (25.0 mg/kg, consecutive 4 times every 3 h) or vehicle were histologically examined. Tissue sections were stained with hematoxylin and eosin. No gross pathophysiological changes were apparent in any of the tissues examined. Scale bars, 200 μm. Source data are provided as a Source Data file.

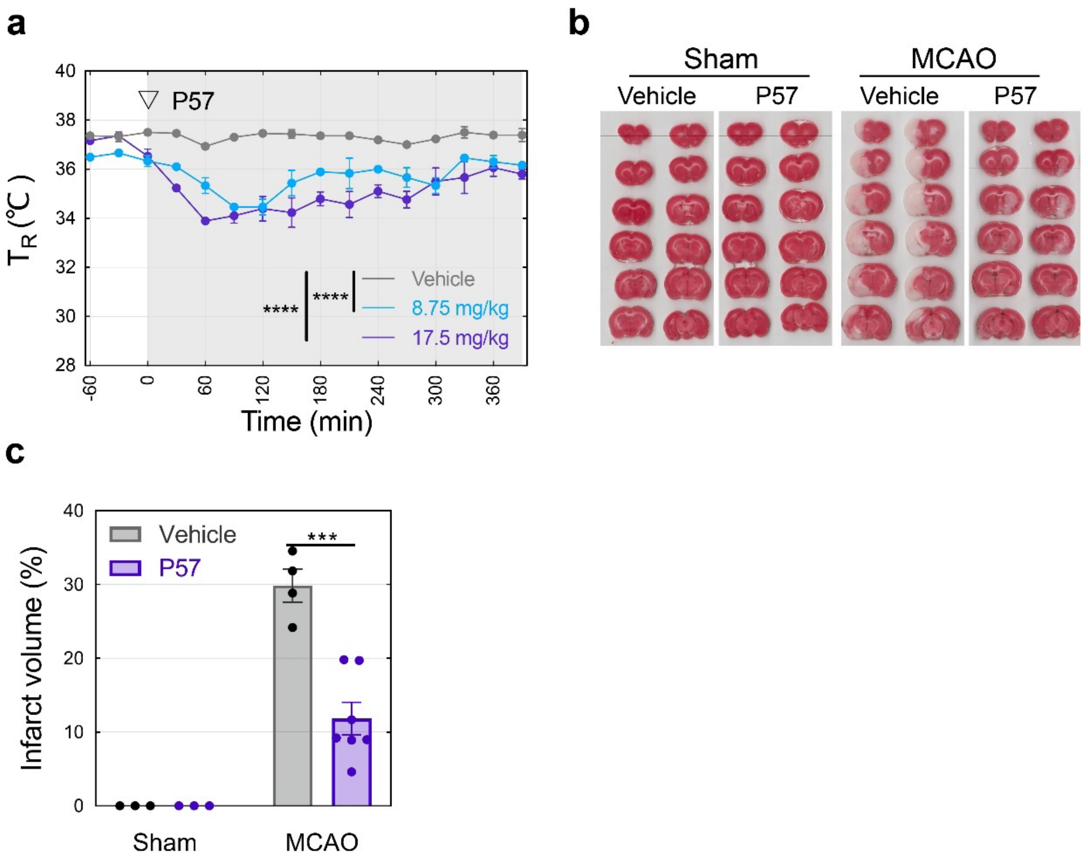

Fig. S3 P57 has a neuroprotective effect in MCAO model. a, Rectal temperature of P57-treated Wistar rats. P57 (8.8 mg/kg or 17.5 mg/kg) or the vehicle was injected intraperitoneally into rats at 0 min (arrow). T_R was measured every 30 minutes, mean (n = 3 mice) ± s.e.m; **** P < 0.0001 compared to control group, as determined by student's t test. **b,** Representative images of TTC staining of brain slices in MCAO model treated with P57. P57 (17.5 mg/kg) or the vehicle was injected intraperitoneally into rats 2 h after middle cerebral artery occlusion. Sham represents sham-operated group, in which Wistar rats underwent all of the operative process except the middle cerebral artery occlusion (MCAO). **c,** Statistic infarct volumes of brain slices. Mean (n = 7 mice) ± s.e.m; *** P < 0.001 compared to MCAO-operated control group, as determined by student's t test. Experiments in fig.S3 was performed under ambient temperatures at 22~24℃. Student's t test used was two-sided. Source data are provided as a Source Data file.

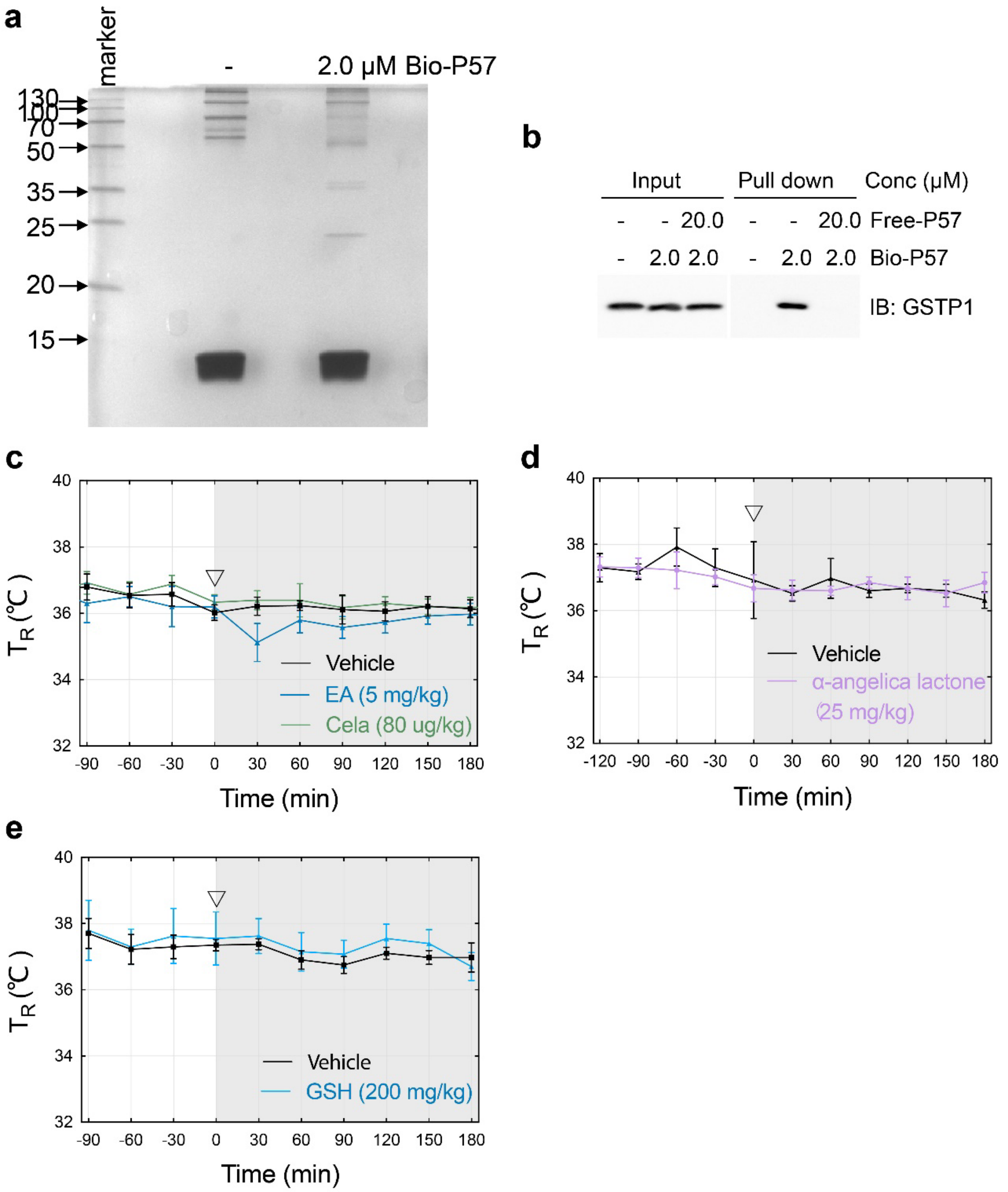

Fig. S4 P57 interacts with PDXK. a, Affinity chromatography experiment with mouse brain lysate using P57-Bio (2.0 μM) probe. Silver staining was performed to measure the binding proteins with P57-Bio. **b,** Affinity chromatography experiment with mouse brain lysate using P57-Bio (2.0 μM) probe in the absence (middle lane) and presence (right lane) of P57 (20.0 μM). Western blot was performed to measure the binding amount of GSTP1 with P57-Bio. **c,** Core temperature of EA or Cela-treated mice. EA (5.0 mg/kg),

Cela (80.0 μg/kg) or the vehicle was injected intraperitoneally into mice at 0 min (arrow). T_R was measured every 30 minutes, mean (n = 5 mice) ± s.e.m. **d,** Core temperature of α-angelica lactone-treated mice. α-angelica lactone (25.0 mg/kg) or the vehicle was injected intraperitoneally into mice at 0 min (arrow). T_R was measured every 30 minutes, mean (n = 4 mice) ± s.e.m. **e,** Core temperature of GSH-treated mice. GSH (200.0 mg/kg) or the vehicle was injected intraperitoneally into mice at 0 min (arrow). T_R was measured every 30 minutes, mean (n=4 mice) ± s.e.m. Source data are provided as a Source Data file.

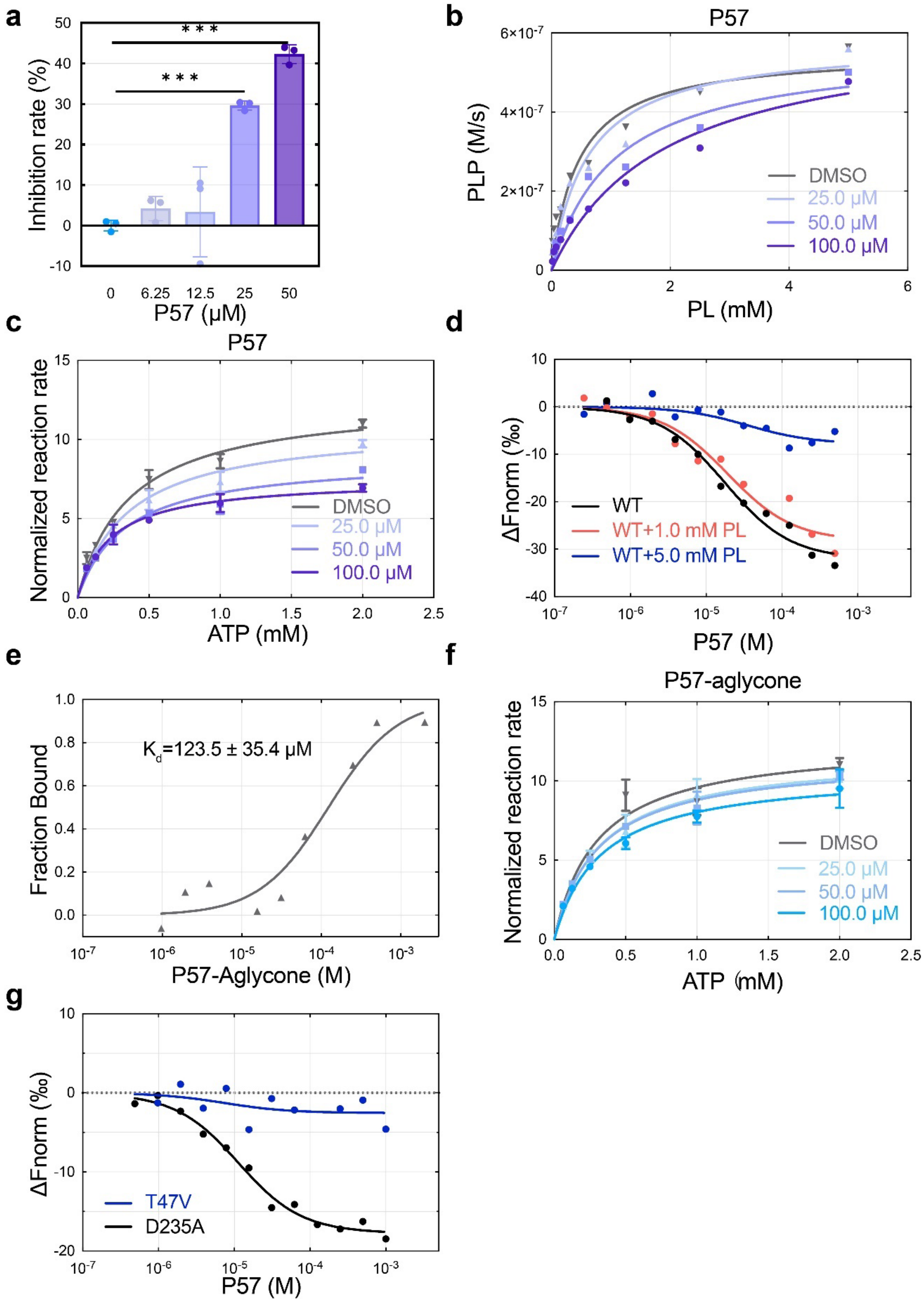

Fig. S5 P57 inhibits enzyme activity of PDXK. a, Effect of P57 on PDXK enzyme activity. The activity

was measured in the standard assay condition at 37°C with various concentrations of P57, mean (n = 3 mice) ± s.e.m; *** $P < 0.001$, as determined by student's t test (two-sided). **b,** Effect of P57 on the human pyridoxal kinase activity. The activity was measured at a fixed concentration of ATP (1.5 mM) and varying concentrations of PL at 37°C with various concentrations of P57. **c,** Effect of P57 on the human pyridoxal kinase activity. The activity was measured at a fixed concentration of PL (300 μM) and varying concentrations of ATP at 37°C with various concentrations of P57. Mean (n = 2 biologically independent samples) ± s.e.m. **d,** Dose-response curve for the competitive binding interaction of P57 and PL with PDXK using MST. The concentration of PDXK is kept constant, while the concentration of P57 varies from 0 to 100 μM in the presence of PL (0 mM, 1.0 mM or 5.0 mM). Notably, the sign of the MST signal amplitude is decreased from 32.1 (without PL) to 7.8 (with 5.0 mM PL). P57 showed a lower affinity of K_d (35.5 μM) in the presence of PL (1.0 mM). **e,** Dose-response curve for the binding interaction between P57-aglycone and PDXK using MST. The concentration of PDXK is kept constant, while the concentration of P57-aglycone varies from 0 to 100 μM. The binding curve yields a dissociation constant $K_d = 123.5 \pm 35.4$ μM. **f,** Effect of P57-aglycone on the human pyridoxal kinase activity. The activity was measured at a fixed concentration of PL (300 μM) and varying concentrations of ATP at 37°C with various concentrations of P57-aglycone. Mean (n = 2 biologically independent samples) ± s.e.m. **g,** The binding of P57 to protein PDXK mutants is quantified using MST. Mutated T47V showed a lower affinity with an undetectable K_d (blue). Mutant D235A showed a comparable affinity of K_d (11.3 μM) (black). Source data are provided as a Source Data file.

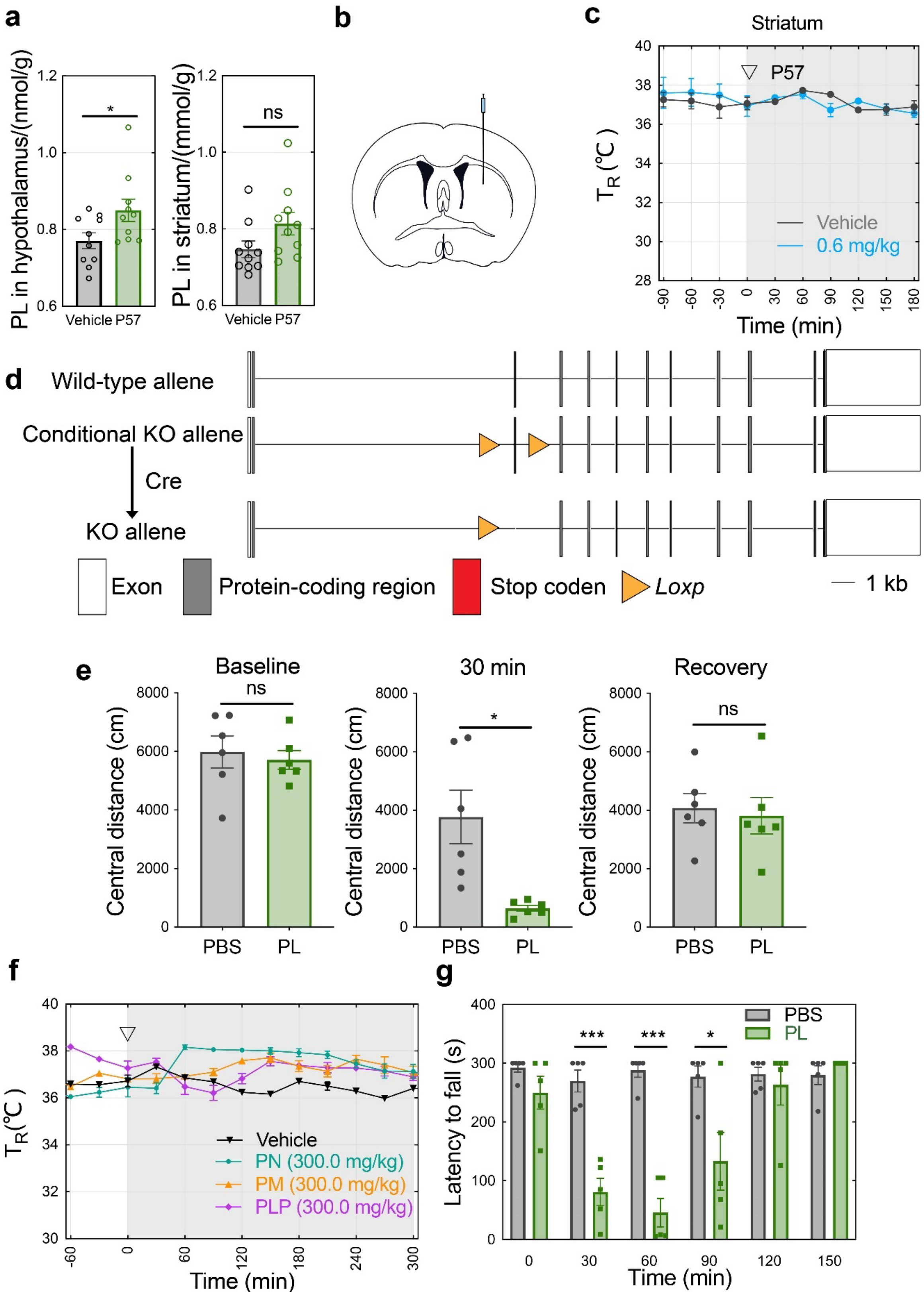

Fig. S6 The effect of PL on thermoregulation and behavior of mice. a, LC-MS detection of PL in

hypothalamus (left) and striatum (right). Hypothalamus and striatum were sampled at 90 min after treated with P57 (25.0 mg/kg), mean (n = 10 mice) ± s.e.m.; * $P < 0.05$ compared to control group, as determined by student's t test. Detection of PL in tissues was confirmed by mass and retention time relative to the pure standard. **b,** Schematic showing the lateral injection of P57 (0.6 mg/kg) into the striatum. **c,** Core temperature of mice treated with P57 in the striatum. P57 (0.6 mg/kg) or the vehicle was injected into the striatum at 0 min (arrow). T_R was measured every 30 minutes, mean (n = 3 mice) ± s.e.m. **d,** Schematics of the wild-type and Loxp site knockin alleles of *Pdxk*. Rectangles indicate exons, and protein-coding regions are shaded gray. Triangles filled with orange represent Loxp. **e,** Travel distance recording in open-field test at baseline before treatment with vehicle or PL, 30 minutes and 24 hours (Recovery) after intraperitoneal administration of vehicle or PL (300.0 mg/kg), mean (n = 6 mice) ± s.e.m; * $P < 0.05$ and *** $P < 0.001$, as determined by student's t test. **f**, Rectal temperature of PN, PM and PLP-treated mice. PN (300.0 mg/kg), PM (300.0 mg/kg), PLP (300.0 mg/kg) or the vehicle was injected intraperitoneally into mice at 0 min (arrow), mean (n = 3 mice) ± s.e.m. **g,** Rotarod performance of PL-treated mice. PL (300.0 mg/kg) or the vehicle control was injected intraperitoneally into mice at 0 min. Latency to fall from a constant accelerating rotarod was recorded at 0, 30, 60, 120 and 150 minutes. Mean (n = 5 mice) ± s.e.m; * $P < 0.05$, as determined by student's t test. Student's t test used was two-sided. Source data are provided as a Source Data file.

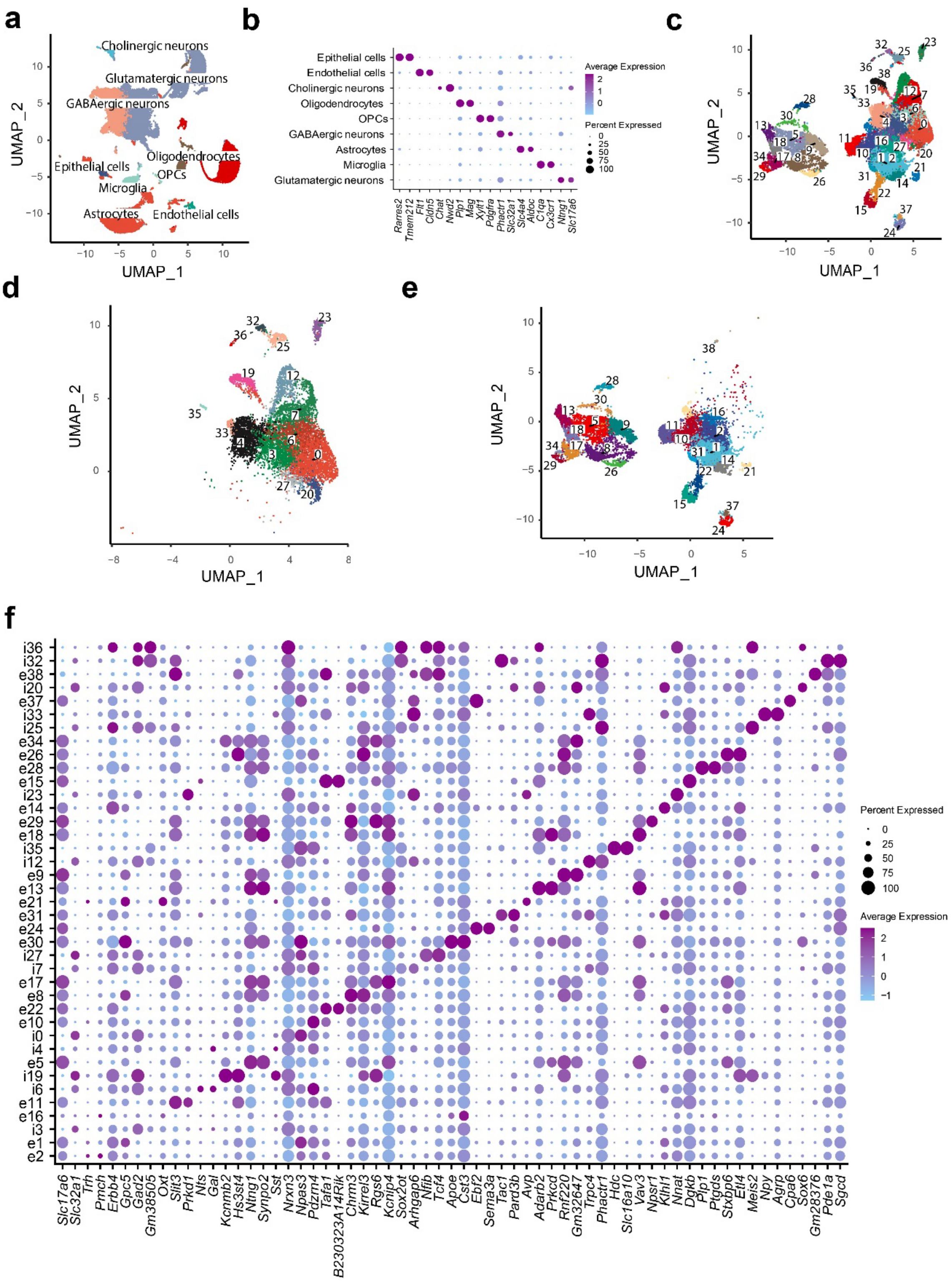

Fig. S7 Overview of the cell clusters in hypothalamus by snRNA-seq. a, UMAP plot from 40,008 nuclei in hypothalamus of 3 groups. Colors are used to distinguish the main cell types. OPCs means the

oligodendrocyte precursor cells. **b,** Expression of the marker genes across different cell types in **a**. **c,** UMAP plot presents 21,411 neuronal nuclei. Colors classify to 39 subtypes. **d, e,** UMAP plots for excitatory and inhibitory neurons in hypothalamus. **d,** 15 GABAergic neuron subtypes; **e,** 24 Glutamatergic neuron subtypes. **f,** Expression of top 2 marker genes across neuronal subtypes. Cell subtypes are named with a numeric following the acronym of cell class (e, excitatory; i, inhibitory). Cell subtypes are organized based on hierarchical clustering.

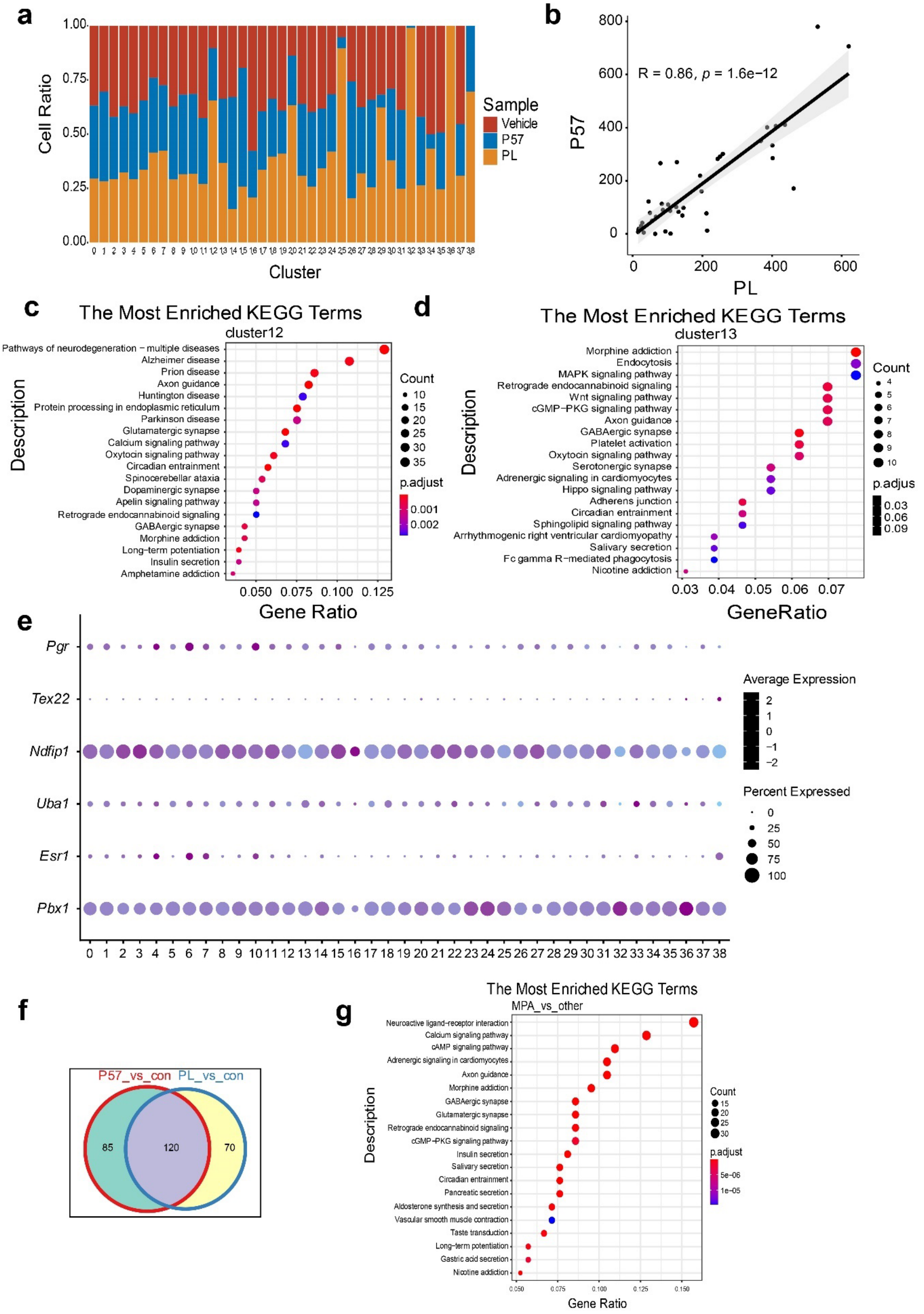
a
Cell Ratio
1.00
0.75
0.50
0.25
0.00
Cluster
Sample
Vehicle
P57
PL
b
R = 0.86, p = 1.6e−12
P57
PL
800
600
400
200
0
c
The Most Enriched KEGG Terms
cluster12
Description
Pathways of neurodegeneration − multiple diseases
Alzheimer disease
Prion disease
Axon guidance
Huntington disease
Protein processing in endoplasmic reticulum
Parkinson disease
Glutamatergic synapse
Calcium signaling pathway
Oxytocin signaling pathway
Circadian entrainment
Spinocerebellar ataxia
Dopaminergic synapse
Apelin signaling pathway
Retrograde endocannabinoid signaling
GABAergic synapse
Morphine addiction
Long−term potentiation
Insulin secretion
Amphetamine addiction
Gene Ratio
0.050
0.075
0.100
0.125
Count
10
15
20
25
30
35
p.adjust
0.001
0.002
d
The Most Enriched KEGG Terms
cluster13
Description
Morphine addiction
Endocytosis
MAPK signaling pathway
Retrograde endocannabinoid signaling
Wnt signaling pathway
cGMP−PKG signaling pathway
Axon guidance
GABAergic synapse
Platelet activation
Oxytocin signaling pathway
Serotonergic synapse
Adrenergic signaling in cardiomyocytes
Hippo signaling pathway
Adherens junction
Circadian entrainment
Sphingolipid signaling pathway
Arrhythmogenic right ventricular cardiomyopathy
Salivary secretion
Fc gamma R−mediated phagocytosis
Nicotine addiction
GeneRatio
0.03
0.04
0.05
0.06
0.07
Count
4
5
6
7
8
9
10
p.adjus
0.03
0.06
0.09
e
Pgr
Tex22
Ndfip1
Uba1
Esr1
Pbx1
0 1 2 3 4 5 6 7 8 9 10 11 12 13 14 15 16 17 18 19 20 21 22 23 24 25 26 27 28 29 30 31 32 33 34 35 36 37 38
Average Expression
2
1
0
−1
−2
Percent Expressed
0
25
50
75
100
f
P57_vs_con
PL_vs_con
85
120
70
g
The Most Enriched KEGG Terms
MPA_vs_other
Description
Neuroactive ligand-receptor interaction
Calcium signaling pathway
cAMP signaling pathway
Adrenergic signaling in cardiomyocytes
Axon guidance
Morphine addiction
GABAergic synapse
Glutamatergic synapse
Retrograde endocannabinoid signaling
cGMP−PKG signaling pathway
Insulin secretion
Salivary secretion
Circadian entrainment
Pancreatic secretion
Aldosterone synthesis and secretion
Vascular smooth muscle contraction
Taste transduction
Long−term potentiation
Gastric acid secretion
Nicotine addiction
Gene Ratio
0.050
0.075
0.100
0.125
0.150
Count
15
20
25
30
p.adjust
5e−06
1e−05

Fig. S8 Evaluation of of P57 and PL-induced transcriptional changes in hypothalamus by snRNA-seq. a, Bar plot showing the cell ratio of each group (control, P57 and PL) in each cluster. **b,** Scatter plot shows the correlation of the number of cells in each cluster between P57 and PL. Each point represents a cluster. The linear best fit line is shown, and the Pearson correlation coefficient (R) and p-value (P) were calculated (two-sided test). The solid black lines with 95% confidence intervals (shaded areas) indicate a linear regression fit. **c, d,** KEGG enrichment analysis of overlapped DEGs between P57 and PL in cluster 12 (**c**) and cluster 13 (**d**). The analysis employed the hypergeometric distribution to calculate the significance p-value (one-sided test) and applied the Benjamini and Hochberg (BH) method for multiple hypothesis testing correction. **e,** Expression of marker gene for MPA (*Pbx1, Esr1, Uba1, Ndfip1, Tex22, Pgr*) in different subtypes. **f**, Venn plot shows the overlap of DEGs of P57 and PL in the MPA compared with control group, respectively. **g,** KEGG pathway enrichment analysis of DEGs in the MPA and all other neurons. P-value was calculated by the hypergeometric distribution (one-sided test), and applied the Benjamini and Hochberg (BH) method for multiple hypothesis testing correction.

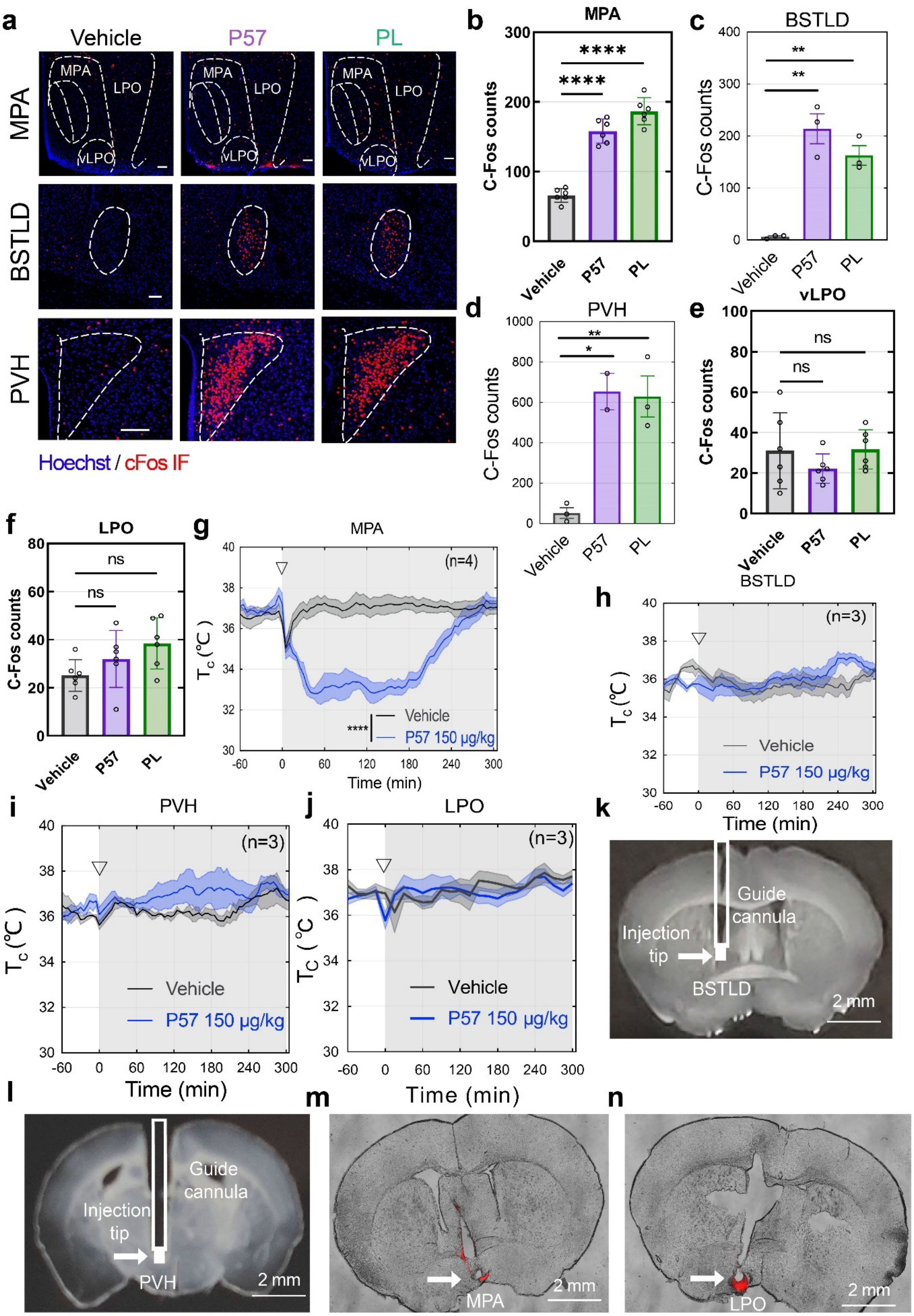
a
Vehicle
P57
PL
MPA
BSTLD
PVH
MPA
LPO
vLPO
Hoechst / cFos IF
b
MPA
C-Fos counts
Vehicle
P57
PL
****
c
BSTLD
**
d
PVH
*
e
vLPO
ns
f
LPO
g
MPA
(n=4)
T_c (°C)
Time (min)
Vehicle
P57 150 μg/kg
h
BSTLD
(n=3)
i
PVH
j
LPO
k
Guide cannula
Injection tip
2 mm
l
m
n

Fig. S9 Both P57 and PL activate neurons in MPA, BSTLD and PVH. a, Brain images containing POA, BSTLD and PVH immunostained with a neuronal activation marker (c-Fos) 160 min after intraperitoneal injection of vehicle, P57 (25.0 mg/kg) or PL (300.0 mg/kg) (representative of n = 6 mice for MPA and n = 3 mice for BSTLD and PVH). Scale bars, 100 μm. The dashed white lines indicate boundaries between subregions. MPA, medial preoptic area; BSTLD, bed nucleus of the stria terminalis, lateral division, dorsal part; PVH, paraventricular hypothalamus. **b, c, d, e, f,** Quantification of c-Fos-positive neurons in the MPA (b),BSTLD (c), PVH (d), vLPO (e) and LPO (f) of each group of (**a**). Dots represent the raw values in each group. n = 6 mice for MPA, vLPO and LPO and n = 3 mice for BSTLD and PVH, one-way ANOVA. LPO, lateral preoptic nucleus; vLPO, ventrolateral preoptic nucleus. **g, h, i, j,** Core temperature of mice treated with P57 in the MPA (g), BSTLD (**h**), PVH (**i**) and LPO (**j**). P57 (150 μg/kg) or the vehicle control was injected into the MPA, BSTLD, PVH or LPO at 0 min (arrow). Core temperature (T_c) was measured and recorded by Anilogger® core temperature monitoring system every 15 minutes, n= 4 for MPA group, n = 3 mice for others, two-way ANOVA. **k**, **l,** Coronal brain sections showing location of cannula implanted in BSTLD (**k**) and PVH (**l**). Scale bars, 2 mm. **m, n,** Coronal brain sections showing location of cannula implanted in MPA (**m**) and LPO (**n**). Mice brain was fixed at 120 min after 200 nL Dil injected into the MPA or LPO to demonstrate placement for injection and show the diffusion range of P57 or the vehicle. Red shows the 200 nL Dil diffusion range. Scale bars, 2 mm. All error bars are presented as mean values ± s.e.m. * P < 0.05, ** P < 0.01, **** P < 0.0001 and ns, not significant. Source data are provided as a Source Data file.

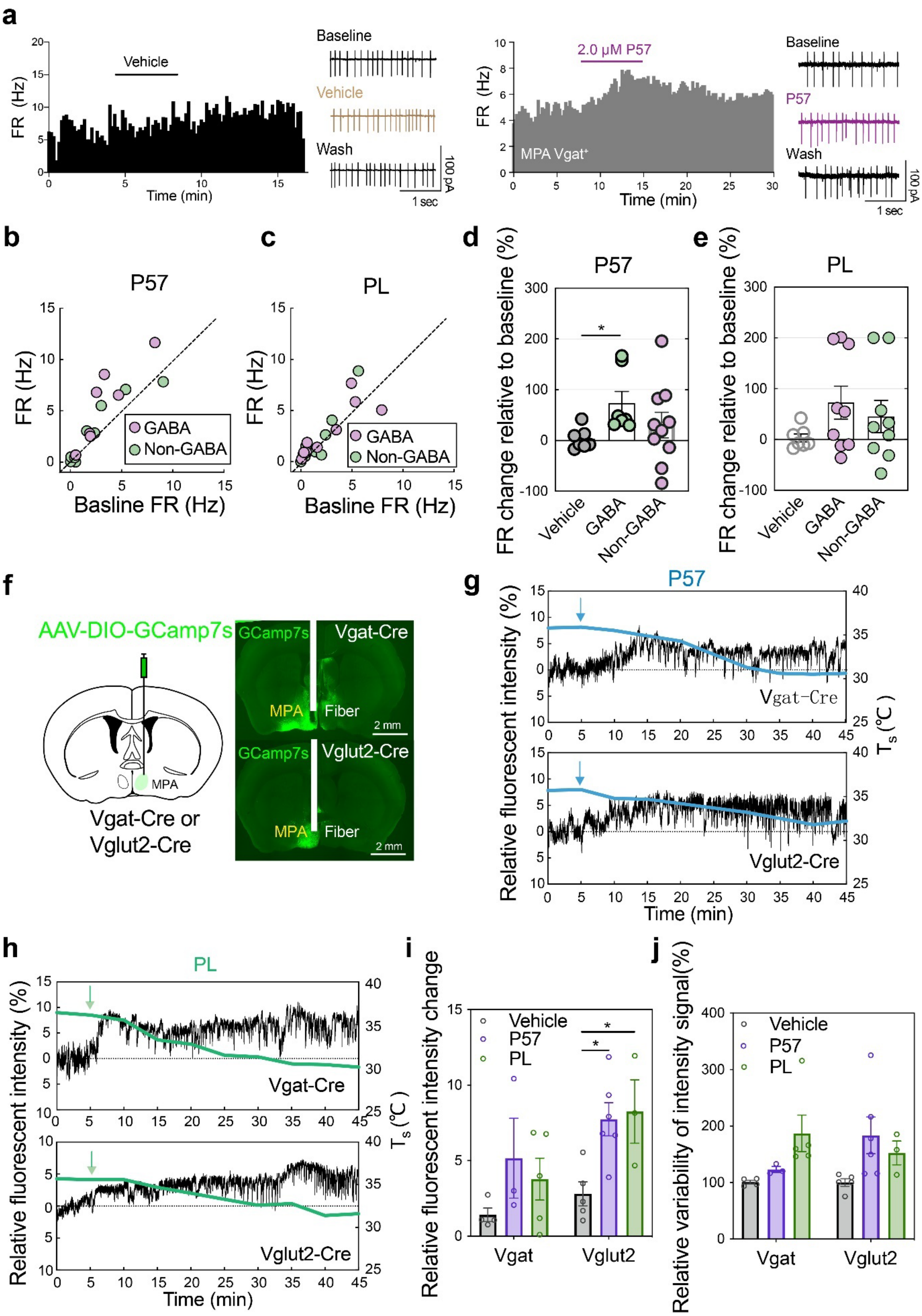
a
Vehicle
FR (Hz)
Time (min)
Baseline
Vehicle
Wash
100 pA
1 sec
2.0 μM P57
MPA Vgat+
FR (Hz)
Time (min)
Baseline
P57
Wash
100 pA
1 sec
b
P57
FR (Hz)
Basline FR (Hz)
GABA
Non-GABA
c
PL
FR (Hz)
Basline FR (Hz)
GABA
Non-GABA
d
P57
FR change relative to baseline (%)
Vehicle
GABA
Non-GABA
e
PL
FR change relative to baseline (%)
Vehicle
GABA
Non-GABA
f
AAV-DIO-GCamp7s
MPA
Vgat-Cre or
Vglut2-Cre
GCamp7s
Vgat-Cre
MPA
Fiber
2 mm
GCamp7s
Vglut2-Cre
MPA
Fiber
2 mm
g
P57
Relative fluorescent intensity (%)
Vgat-Cre
Vglut2-Cre
Ts (℃)
Time (min)
h
PL
Relative fluorescent intensity (%)
Vgat-Cre
Vglut2-Cre
Ts (℃)
Time (min)
i
Relative fluorescent intensity change
Vehicle
P57
PL
Vgat
Vglut2
j
Relative variability of intensity signal(%)
Vehicle
P57
PL
Vgat
Vglut2

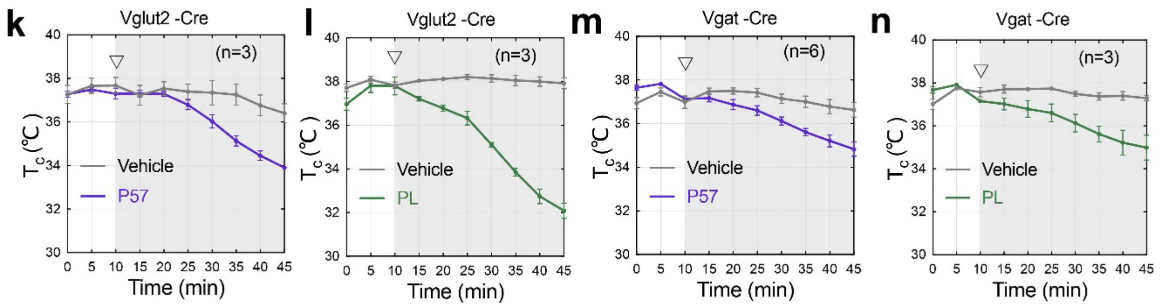

Fig. S10 Effect of both P57 and PL on the activities of neurons in MPA. a, Representative trace of voltage-clamp recording from tdTomato-positive neurons in a brain slice prepared from Vgat-Cre; Ai14 mice (left). We performed the experiments from 7 mice and obtained the same results. Representative comparison of fire frequency at baseline and after treatment with vehicle (right up) or P57 (2.0 μM) (right down). **b,** Firing frequency of GABAergic (n = 7) and non-GABAergic (n = 10) neurons in MPA after treatment with P57 (2.0 μM). Brain slices were prepared from Vgat-Cre; Ai14 mice. Pink dots represent the raw values in GABAergic neurons, green dots represent the raw values in non-GABAergic neurons. **c,** Firing frequency of GABAergic (n = 9) and non-GABAergic (n = 9) neurons in MPA after treatment with PL (10.0 μM). **d,** Relative change of fire frequency of GABAergic and non-GABAergic neurons in brain slices to baseline after treatment with P57 (2.0 μM), n= 7 mice for vehicle and GABA group, n= 10 mice for Non-GABA group, student's t test (two-sided). Brain slices were prepared from Vgat-Cre; Ai14 mice. Pink dots represent the raw values in GABAergic neurons, green dots represent the raw values in non-GABAergic neurons. **e,** Relative change of fire frequency of GABAergic and non-GABAergic neurons in brain slices to baseline after treatment with PL (10.0 μM), n= 7 mice for vehicle, n= 9 mice for GABA group and Non-GABA group. **f,** Schematic showing the injection of AAV2/9-hSyn-DIO-jGCaMP7s-WPRE-pA into the MPA (left) and coronal brain sections showing jGCaMP7s specifically expressed in GABAergic (upper right) or glutamatergic neurons (lower right) in MPA and the location of optical fiber (right). Scale bars, 2 mm. **g, h,** Recording sessions in Vgat-Cre and Vglut2-Cre mice showing Tc and the normalized jGCaMP7s signal. Relative fluorescent intensity is calculated by dividing the smoothed calcium-dependent jGCaMP7s signal with the Ca2+-independent scaled fit. **g,** Example 45-min trace spanning before and after intraperitoneal injection of P57 (25.0 mg/kg) (representative of n = 4 mice). **h,**

Example 45-min trace spanning before and after intraperitoneal injection of PL (300.0 mg/kg) (representative of n = 3 mice). **i,** Relative fluorescent intensity changes of jGCaMP7s signal recordings of Vglut2-Cre (n = 5) or Vgat-Cre (n = 5) mice with respective treatment of P57 (25.0 mg/kg) and PL (300.0 mg/kg), student's t test (two-sided). **j,** Relative variability of jGCaMP7s signal recordings of Vglut2-Cre (n = 5) or Vgat-Cre (n = 5) mice with respective treatment of P57 (25.0 mg/kg) and PL (300.0 mg/kg). **k**, **m**, Core temperature of P57-treated Vglut2-Cre (**k**) or Vgat-Cre mice (**m**). **l**, **n**, Core temperature of PL- treated Vglut2-Cre (**l**) or Vgat-Cre mice (**n**). Core temperature (T_c) was measured and recorded by Anilogger® core temperature monitoring system every 5 minutes at the same time when jGCaMP7s signal was detected, P57 (25 mg/kg), PL (300 mg/kg) or the vehicle was injected intraperitoneally into mice at 0 min (arrow), n= 3 for Vglut2-Cre mice and n= 6 for Vgat-Cre mice. All error bars are presented as mean values ± s.e.m. * $P < 0.05$. Source data are provided as a Source Data file.

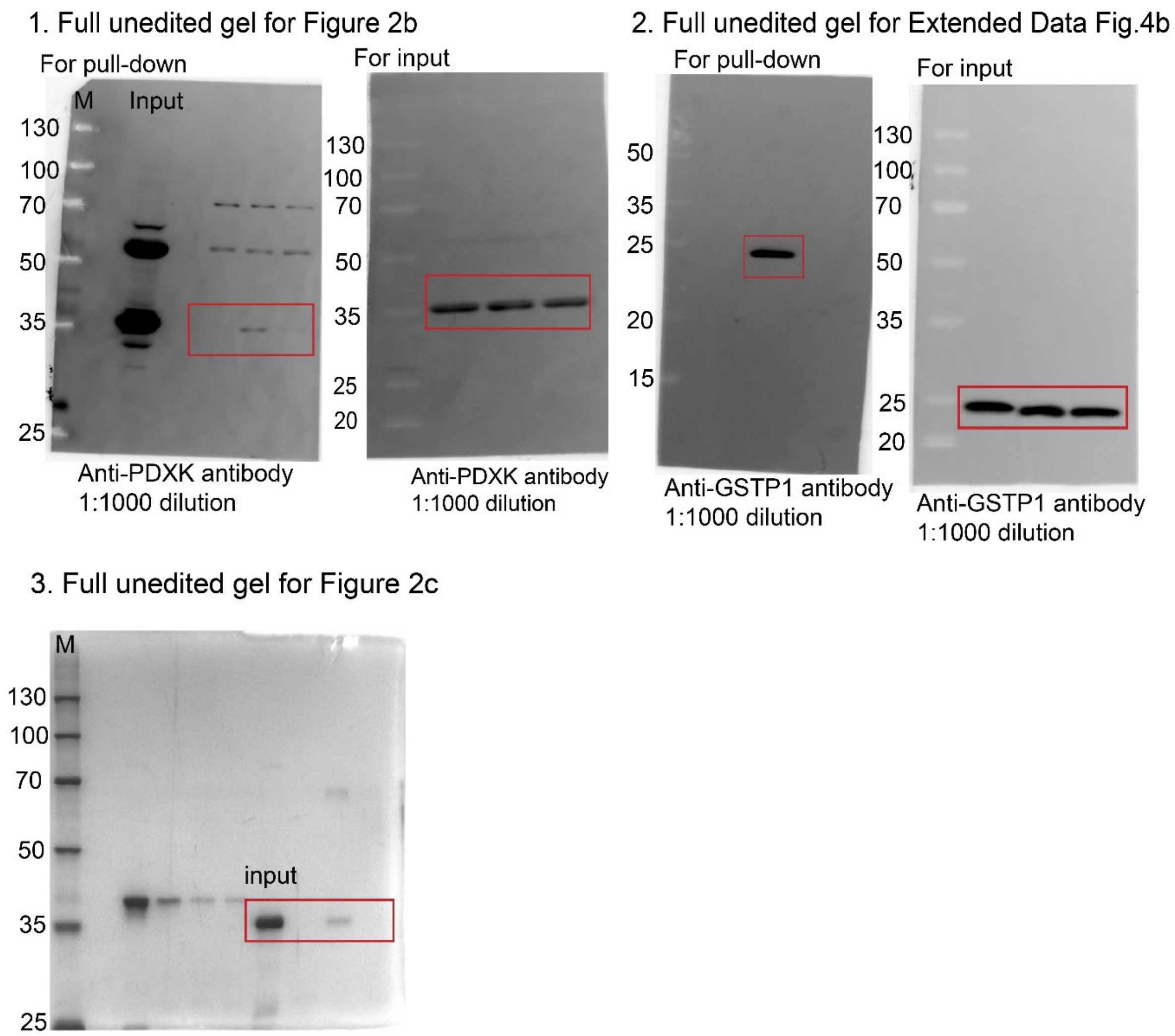

Fig. S11 Full unedited gels shown in Figures and Extended Data Figures.

Table S1. Identified proteins by Mass spectrum of P57 pull-down assay

		Control		P57-biotin	
Protein ID	Protein name	Score	Sequence Coverage	Score	Sequence Coverage
P19157	GSTP1	0	0	584	59%
Q8K183	pyridoxal kinase	119	12%	568	39%
P06837	neuromodulin	0	0	411	66%
A6ZI44	Fructose-bisphosphate aldolase	0	0	196	25%
P35802	Neuronal membrane glycoprotein M6-a	0	0	190	17%
Q7TQD2	Tubulin polymerization-promoting protein	0	0	159	35%
D3YUQ9	Elongation factor 1-delta	0	0	139	29%
P14152	Malate dehydrogenase, cytoplasmic	0	0	136	16%
P08226	Apolipoprotein E	0	0	133	16%
B1AQW2	Microtubule-associated protein	0	0	132	25%
Q8R191	Synaptogyrin-3	0	0	121	19%
B1AQW2	Microtubule-associated protein	0	0	105	18%
Q9DCJ1	Target of rapamycin complex subunit LST8	0	0	105	12%
P47754	F-actin-capping protein subunit alpha-2	0	0	100	16%

Table S2. UPLC-MS/MS-based quantification of B6 vitamers of brain in C57BL mice.

Relative content (%)	n	PN	PM	PL	4-PA	PLP
Vehicle	12	99.84 ± 18.17	99.83 ± 10.51	99.99 ± 32.23	99.99 ± 13.33	100.00 ± 7.29
P57(30 min)	12	74.24 ± 6.13	94.51 ± 4.60	94.46 ± 32.97	98.30 ± 36.69	99.50 ± 9.32
p-value		***	ns	ns	ns	ns
P57(90 min)	12	68.00 ± 8.02	90.00 ± 9.65	99.23 ± 25.92	118.22 ± 19.35	102.09 ± 7.06
p-value		***	*	ns	*	ns

Mean (n = 12 mice) ± s.e.m.; *P<0.05; ***P<0.001; ns, not significant compared to vehicle, as determined by student's t test (two-sided).

Table S3. UPLC-MS/MS-based quantification of B6 vitamers of hypothalamus in C57BL mice.

Relative content (%)	n	PN	PM	PL	4-PA	PLP
Vehicle	12	100.04 ± 19.88	99.86 ± 6.78	99.99 ± 14.24	99.97 ± 14.03	100.00 ± 6.20
P57(30 min)	12	95.28 ± 13.90	104.08 ± 9.27	125.45 ± 26.12	97.15 ± 18.14	101.88 ± 7.90
p-value		ns	ns	**	ns	ns
P57(90 min)	12	97.18 ± 20.51	99.65 ± 9.19	144.28 ± 21.67	93.21 ± 16.95	110.21 ± 11.07
p-value		ns	ns	***	ns	*

Mean (n = 12 mice) ± s.e.m.; *P<0.05; **P<0.01; ***P<0.001; ns, not significant compared to vehicle, as determined by student's t test (two-sided).
