# Supplementary material for: Natural product P57 induces hypothermia through targeting pyridoxal kinase
Source: Nat Commun. 2023 Sep 26;14:5984. doi: 10.1038/s41467-023-41435-y (PMC10522591; doi:10.1038/s41467-023-41435-y)
Supplement: Supplementary file 3 — Reporting Summary [file 41467_2023_41435_MOESM3_ESM.pdf]

nature portfolio

Corresponding author(s): Yongjun Dang

Last updated by author(s): Sep 8, 2023

Reporting Summary

Nature Portfolio wishes to improve the reproducibility of the work that we publish. This form provides structure for consistency and transparency in reporting. For further information on Nature Portfolio policies, see our Editorial Policies and the Editorial Policy Checklist.

Statistics

For all statistical analyses, confirm that the following items are present in the figure legend, table legend, main text, or Methods section.

n/a	Confirmed	
☐	☒	The exact sample size (*n*) for each experimental group/condition, given as a discrete number and unit of measurement
☐	☒	A statement on whether measurements were taken from distinct samples or whether the same sample was measured repeatedly
☐	☒	The statistical test(s) used AND whether they are one- or two-sided<br>*Only common tests should be described solely by name; describe more complex techniques in the Methods section.*
☐	☒	A description of all covariates tested
☐	☒	A description of any assumptions or corrections, such as tests of normality and adjustment for multiple comparisons
☐	☒	A full description of the statistical parameters including central tendency (e.g. means) or other basic estimates (e.g. regression coefficient) AND variation (e.g. standard deviation) or associated estimates of uncertainty (e.g. confidence intervals)
☐	☒	For null hypothesis testing, the test statistic (e.g. *F*, *t*, *r*) with confidence intervals, effect sizes, degrees of freedom and *P* value noted<br>*Give P values as exact values whenever suitable.*
☒	☐	For Bayesian analysis, information on the choice of priors and Markov chain Monte Carlo settings
☐	☒	For hierarchical and complex designs, identification of the appropriate level for tests and full reporting of outcomes
☐	☒	Estimates of effect sizes (e.g. Cohen's *d*, Pearson's *r*), indicating how they were calculated

Our web collection on statistics for biologists contains articles on many of the points above.

Software and code

Policy information about availability of computer code

Data collection	animal temperature measuring apparatus (FT3400, Kew Basis), IR digital thermographic camera (FLIR T430sc), Anilogger® core temperature monitoring system, Ethovision XT video tracking software system (Noldus Information Technologies, Leesburg, VA, USA).
Data analysis	The raw data were analyzed by Mascot search engine (v2.3.0) against the UniProt Mus musculus database (downloaded 2013). Docking studies were performed with methods as previously published (https://doi.org/10.1016/j.ejmech.2019.111767) using Schrödinger suite (version 2009).<br>For sn-seq analysis, Raw data quality (Fastq format) was assessed and poor-quality reads were removed using fastp (version 0.19.5) program. Cleaned data were then mapped to reference genome (mm10) using the default parameters of Cell Ranger v6.0.2 (10X Genomics).<br>Seurat (version 4.0) was used to process and cluster cells.<br>Cell type annotation was performed with SingleR (version 1.6.1). We excluded total 965 cells using the default parameters of the R package DoubletFinder (version 2.0) from three groups (P57, PL, and control). For visualization and analysis and to eliminate batch effects, the three groups were integrated as a Seurat object using Harmony (version 0.1.0). The DEGs between different groups was calculated by using FindMarker function.<br>To explore the potential function of marker genes in different cell populations or DEGs in different groups, we used R package clusterProfiler (version 4.0.5) to perform KEGG Function enrichment analysis. The org.Mm.eg.db package (3.14.0) was used to set the background gene list as all genes detected in sequencing.<br>GraphPad Prism 8.0.1, ImageJ-win64,.

For manuscripts utilizing custom algorithms or software that are central to the research but not yet described in published literature, software must be made available to editors and reviewers. We strongly encourage code deposition in a community repository (e.g. GitHub). See the Nature Portfolio guidelines for submitting code & software for further information.

Data

Policy information about availability of data

All manuscripts must include a data availability statement. This statement should provide the following information, where applicable:

- Accession codes, unique identifiers, or web links for publicly available datasets
- A description of any restrictions on data availability
- For clinical datasets or third party data, please ensure that the statement adheres to our policy

The mass spectrometry data have been deposited to the iProX consortium with the dataset ID IPX0005823000 (URL: https://www.iprox.cn/page/PSV023.html;?url=1675073267636rwMF. Password: Cd0j). The mass spectrometry raw data were analyzed by Mascot search engine (v2.3.0) against the UniProt Mus musculus database (downloaded 2013). Other source date are provided with this paper.

Human research participants

Policy information about studies involving human research participants and Sex and Gender in Research.

Reporting on sex and gender	N/A.
Population characteristics	N/A.
Recruitment	N/A.
Ethics oversight	N/A.

Note that full information on the approval of the study protocol must also be provided in the manuscript.

Field-specific reporting

Please select the one below that is the best fit for your research. If you are not sure, read the appropriate sections before making your selection.

☒ Life sciences ☐ Behavioural & social sciences ☐ Ecological, evolutionary & environmental sciences

For a reference copy of the document with all sections, see nature.com/documents/nr-reporting-summary-flat.pdf

Life sciences study design

All studies must disclose on these points even when the disclosure is negative.

Sample size	No statistical methods were used to predetermine sample size. Suitable sample sizes were estimated based on previous experiments (e.g. Xiao L et al 2018) and are similar to those employed in the field.
Data exclusions	No data were excluded from the analyses.
Replication	All attempts at replication were successful. And the information about the times of each experiment repeated was reported in the Statistics and Reproducibility section.
Randomization	Animals used were randomly assigned to groups before experiments.
Blinding	The investigators were not blinded to allocation during and outcome assessment. Because all the indicators detected in our experiment, such as mouse body temperature, metabolic rate, blood pressure, heart rate, field test, rotary rod test, etc., were directly recorded by the instrument, we just sorted them out and carried out statistical analysis. All data results are presented completely, and there is no subjective tendency in the experiment and data processing.

Reporting for specific materials, systems and methods

We require information from authors about some types of materials, experimental systems and methods used in many studies. Here, indicate whether each material, system or method listed is relevant to your study. If you are not sure if a list item applies to your research, read the appropriate section before selecting a response.

Materials & experimental systems

n/a	Involved in the study	
[ ]	[x]	Antibodies
[x]	[ ]	Eukaryotic cell lines
[x]	[ ]	Palaeontology and archaeology
[ ]	[x]	Animals and other organisms
[x]	[ ]	Clinical data
[x]	[ ]	Dual use research of concern

Methods

n/a	Involved in the study	
[x]	[ ]	ChIP-seq
[x]	[ ]	Flow cytometry
[x]	[ ]	MRI-based neuroimaging

Antibodies

Antibodies used	The following antibodies were used for immunoblotting: PDXK (1:1000; 15309, Proteintech), GSTP1 (1:1000, 15902, Proteintech) and GAP43 (1:1000, 16971, Proteintech). The following antibodies were used for immunostaining: c-FOS (1:1000; 5348S, Cell Signaling). Goat anti-Rabbit 647 (1:1000, A21244, Thermofisher), Peroxidase AffiniPure Goat Anti-Rabbit IgG (H+L) (1:10000, 111-035-003, Jackson ImmunoResearch Inc).
Validation	All the primary antibodies were showed the validation statements on the manufacture's website and validated by our western blot data.

Animals and other research organisms

Policy information about studies involving animals; ARRIVE guidelines recommended for reporting animal research, and Sex and Gender in Research

Laboratory animals	We used 6~8-week-old WT male C57BL/6J mice (SLAC company), Wistar rats (SLAC company), Vglut2-ires-Cre (Jackson Lab), Vgat-ires-Cre (Jackson Lab). All mouse lines are in a WT (C57BL/6J) background.
Wild animals	No wild animals were used in the study.
Reporting on sex	Only male mice or rats were used for temperature measurement.
Field-collected samples	No field collected samples were used in the study.
Ethics oversight	All animal care and experimentation were ethically performed according to procedures approved by the Institutional Animal Care and Use Committee at Fudan University.

Note that full information on the approval of the study protocol must also be provided in the manuscript.
